# Veno-Arterial Extracorporeal Membrane Oxygenation in Cardiotoxic Drug-Induced Cardiogenic Shock: A Systematic Narrative Review

**DOI:** 10.3390/life15060925

**Published:** 2025-06-09

**Authors:** Debora Emanuela Torre, Domenico Mangino, Carmelo Pirri

**Affiliations:** 1 Department of Cardiac Anesthesia and Intensive Care Unit, Cardiac Surgery, Ospedale Dell’Angelo, 30174 Venice Mestre, Italy; 2 Cardiac Surgery Department, Ospedale Dell’Angelo, 30174 Venice Mestre, Italy; domenico.mangino@aulss3.veneto.it; 3 Department of Neurosciences, Institute of Human Anatomy, University of Padova, 35121 Padova, Italy; carmelo.pirri@unipd.it

**Keywords:** extracorporeal membrane oxygenation, drug-induced shock, toxic cardiomyopathy, refractory cardiogenic shock, hemodynamic support, extracorporeal life support, poisoning-related cardiovascular failure

## Abstract

**Background:** Severe poisoning can lead to catastrophic cardiovascular collapse, often progressing to multiorgan failure and death. While intensive supportive care and pharmacological intervention remain the cornerstone of management, cases of refractory cardiogenic shock, particularly those caused by membrane stabilizing agents and calcium channel blockers, pose a significant therapeutic challenge. Extracorporeal membrane oxygenation (ECMO) has emerged as a potential life-saving intervention in critically ill patients. This review examines the feasibility, clinical outcomes, and optimal indications for ECMO in the management of drug-induced cardiogenic shock. **Methods**: A systematic narrative review was conducted to evaluate the current evidence of ECMO use in poisoning-related cardiovascular failure, with a particular focus on patient selection criteria and the prognostic determinants of therapeutic resistance. **Results**: Extracorporeal membrane oxygenation may serve as a crucial hemodynamic support strategy in drug-induced circulatory collapse. Most reported cases involve peripheral ECMO, demonstrating variable but promising survival outcomes. **Conclusions**: Despite its potential to rescue patients from otherwise fatal toxic cardiomyopathy, the role of ECMO remains incompletely defined. Further prospective studies are essential to refine patient selection criteria and identify the toxicant-specific predictors of therapeutic failure. A deeper understanding of these factors may enhance clinical decision making and improve survival rates in severe poisoning cases.

## 1. Introduction

The progression of organ failure is a major determinant of mortality in patients with acute poisoning. Over the years, advances in critical care have significantly improved survival in cases of toxicant-induced organ dysfunction. Endotracheal intubation and mechanical ventilation have markedly enhanced outcomes in sedative-induced respiratory failure, while renal replacement therapy has played a pivotal role in mitigating the lethality of toxicant-related acute kidney injury. In selected cases, even fulminant hepatic failure can be successfully managed through liver transplantation.

Cardiotoxic agents remain a leading cause of drug-related mortality [1]. Among them, calcium channel blockers (CCBs) and beta blockers (BBs) rank as the sixth and seventh most frequent causes of fatal drug intoxications, respectively [2]. The primary pathophysiological mechanism underlying cardiotoxicity is the suppression of myocardial contractility, mediated by the inhibition of membrane channels and/or receptors within the conduction system and myocardial tissue. This results in severe cardiac dysfunction, manifesting as impaired conduction and/or contractility, leading to bradycardia, hypotension, peripheral organ hypoperfusion and, in the most severe cases, cardiac arrest. The latter represents the most catastrophic outcome, particularly when refractory to conventional cardiopulmonary resuscitation (CPR), and is associated with a poor prognosis.

In cases of drug-induced severe cardiac failure, extracorporeal life support (ECLS), particularly veno-arterial extracorporeal membrane oxygenation (V-A ECMO), has emerged as a viable therapeutic strategy to maintain circulatory and pulmonary functions while allowing for spontaneous drug clearance and myocardial recovery [3,4,5]. Although its use in toxicological emergencies remains rare (reported in only 0.0004% of cases in a multicenter registry), ECLS has demonstrated efficacy in stabilizing patients with profound cardiotoxicity, including those experiencing refractory cardiac arrest, with relatively favorable outcomes [6]. However, the precise indications, optimal timing for initiation, and overall prognostic impact of ECLS in cardiotoxicant-related poisoning remain poorly defined due to the heterogeneity of toxic agents involved, the frequent presence of multiple co-intoxicants, and the lack of randomized clinical trials.

This review aims to critically evaluate the indications and timing for ECLS initiation, as well as the adjunctive supportive therapies commonly employed in conjunction with ECLS.

## 2. Materials and Methods

This systematic narrative review aims to explore the role of extracorporeal life support in cardiogenic shock induced by cardiotoxic drugs. A comprehensive literature search was performed in the PubMed and Scopus databases, targeting articles published between 1992 and 2025. The following search terms were used: “Extracorporeal membrane oxygenation cardiotoxicity”, “Extracorporeal membrane oxygenation drug poisoning” and “Extracorporeal life support cardiotoxicity”, yielding a total of 406 papers. Although the structure and synthesis followed a narrative approach, predefined inclusion and exclusion criteria were applied to enhance methodological transparency and rigor. This review focuses on studies reporting clinical data on adult or pediatric patients who developed cardiogenic shock as a direct result of exposure to cardiotoxic pharmacological agents, such as beta blockers, calcium channel blockers, tricyclic antidepressants, and antiarrhythmics, and were subsequently treated with ECLS, including veno-arterial extracorporeal membrane oxygenation (V-A ECMO).

**Figure 1:**
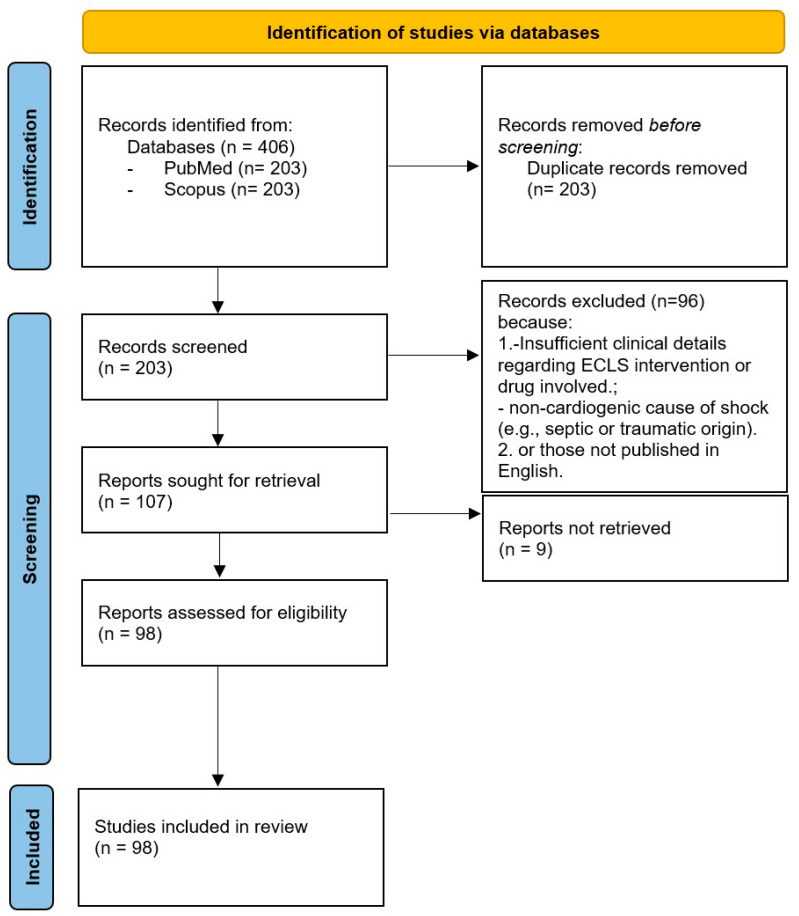
Flow chart of study selection.

Eligible sources included observational studies (prospective or retrospective), case series with at least three patients, and well-documented case reports describing the use of ECLS in the context of cardiotoxic drug poisoning. Systematic reviews and meta-analyses were screened to identify additional relevant primary studies. Outcomes of interest included survival, recovery of cardiac function, duration of extracorporeal support, ECLS-related complications, and pharmacokinetics parameters, when available. Exclusion criteria comprised studies addressing non-cardiogenic forms of shock (e.g., septic, distributive, or hypovolemic), cases of cardiogenic shock unrelated to pharmacological causes (e.g., acute coronary syndromes, viral myocarditis, trauma, or congenital heart disease), and poisonings without significant myocardial depression (e.g., paracetamol, methanol, or non-cardiotoxic pesticides). Additionally, experimental studies on animal models or in vitro systems without translational relevance, as well as editorials, commentaries, opinion pieces, letters without original data, and duplicate publications, were excluded. When multiple publications were derived from the same dataset, only the most comprehensive or most recent version was retained. Studies lacking sufficient clinical detail, such as the specific drug involved, the rationale for ECLS initiation, the modality of support, or relevant outcome data, were also excluded. After applying these criteria, 203 articles were initially identified. Following title and abstract screening, 107 were selected for full text review. Ultimately, 98 studies were included based on methodological and scientific quality (Figure 1).

## 3. Results

Poisoning is characterized by cellular injury or death resulting from the exposure to exogenous toxic agents. Each toxic substance disrupts specific molecular pathways essential for cellular function. The management of poisoning encompasses several key strategies, including preventing further exposure, enhancing toxin elimination when feasible, providing comprehensive supportive care, and administering antidotal therapies that either counteract or bypass the toxin’s effects at its molecular target. While certain poisons induce direct cytotoxicity, others transiently impair cellular function to a degree that jeopardizes patient survival. In severe cases, extracorporeal therapies, such as hemodialysis for toxin clearance or veno-arterial extracorporeal membrane oxygenation (VA-ECMO) for cardiovascular support, may be necessary to sustain life and enable recovery (Table 1).

### 3.1. Beta Blockers (BBs)

Beta-adrenergic antagonists are widely prescribed for the management and prevention of cardiovascular disease. Their mechanism of action involves the competitive inhibition of epinephrine and norepinephrine binding to beta-adrenergic receptors, leading to a reduction in myocardial conduction velocity and contractile force.

Toxicity from beta blockers (BBs) represents a major cause of poisoning-related mortality [1]. Severe intoxication results in profound hypotension, primarily driven by bradycardia and diminished myocardial contractility [9]. Additionally, certain beta blockers exert proarrhythmic effects via sodium or potassium channel blockade. Bradycardia is primarily attributed to direct beta-1-adrenergic antagonism, whereas hypotension may stem from a combination of cardiogenic failure, vasodilation secondary to an alpha-1-adrenergic receptor blockade, or other multifactorial mechanisms. In addition, beta blocker poisoning has also been associated with episodes of hypoglycemia, although the underlying pathophysiology remains complex. Hypoglycemia management involves standard dextrose supplementation [10].

**Figure 2:**
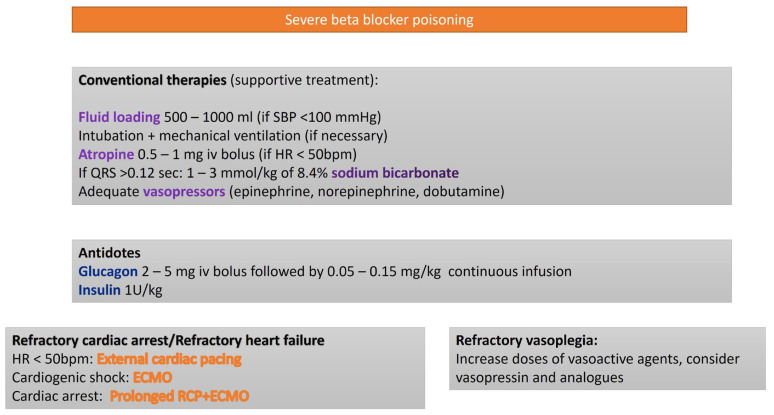
Algorithm for the treatment of severe BB poisoning.

The therapeutic approach to BB poisoning includes high dose insulin therapy [11], atropine [12], glucagon [13], calcium, vasopressors [12], and intravenous lipid emulsion (ILE) therapy [14]. In cases refractory to conventional interventions, veno-arterial extracorporeal membrane oxygenation (VA-ECMO) has been employed as a rescue strategy [15] (Figure 2).

ECMO provides temporary cardiopulmonary support, maintaining systemic perfusion and oxygenation while facilitating the clearance of the offending beta blocker agent. The decision to initiate ECMO should be promptly undertaken within a multidisciplinary team context, considering the patient’s clinical status, anticipated prognosis, and institutional capabilities. Prompt ECMO deployment may be pivotal in mitigating the morbidity and mortality associated with severe BB toxicity. This comprehensive approach underscores the importance of rapid assessment and intervention, integrating advanced pharmacologic strategies with mechanical circulatory support to optimize patient outcomes in life-threatening beta blocker poisoning [16].

In a retrospective study, Friedrichson et al. [7] evaluated the efficacy of V-A ECMO in the management of cardiovascular failure secondary to poisoning with cardiovascular medications. The study included 49 patients, 34.7% of whom had ingested BBs. The overall survival rate with V-A ECMO support was 63.6%. Notably, no statistically significant difference in survival was observed between early and late ECMO initiation.

Similarly, in a single-center cohort study, Vandroux et al. [17] investigated the predictive factors necessitating extracorporeal membrane oxygenation in patients who attempted suicide via the ingestion of cardiac medications, including BBs. The study focused on patients admitted to the intensive care unit with refractory cardiogenic shock requiring ECMO. The key predictors for ECMO necessity included the type and quantity of cardiac medication ingested, the severity of initial hemodynamic compromise, and the patient’s response to conventional resuscitative measures.

A pulse pressure < 35 mmHg or a left ventricular ejection fraction < 20% emerged as the most relevant survival predictors.

Unlike the study by Megarbane et al. [18], which established a lactate cutoff of 3 mmol/L as a prognostic marker, Vandroux et al. did not find lactate levels to be predictive of mortality, likely due to the high prevalence of metformin use in their cohort.

Their findings highlight the importance of the early recognition of these factors to facilitate timely ECMO initiation, potentially improving survival outcomes.

In another study, Voiciu et al. [6] analyzed multiple prospective and retrospective studies to propose indications for ECLS, incorporating their clinical experience. They suggested initiating ECLS preferably within a short duration of resuscitation (<30 min) to optimize survival outcomes. However, they emphasized that ECLS should not be ruled out solely based on prolonged resuscitation, as survival has been reported even after 180 min of cardiopulmonary resuscitation. Additional criteria included persistent cardiac dysfunction despite catecholamine support (LVEF < 40–50%), high-dose vasopressor requirements (epinephrine + norepinephrine + isoproterenol doses > 4 mcg/kg/min), and blood lactate concentration > o = 5 mmol/L.

### 3.2. Calcium Channel Blockers

Calcium channel blockers (CCBs) are classified as Class IV antiarrhythmic agents. Over recent years, they have become the leading cause of cardiotoxicity-related fatalities, largely due to their widespread use in hypertension management, given their efficacy and good tolerability [1]. CCBs are broadly categorized into two pharmacological classes:

- Dihydropyridines (e.g., nifedipine, amlodipine, lercanidipine), which primarily induce peripheral vasodilation with minimal negative inotropic effect.
- Non-dihydropyridines (e.g., diltiazem, verapamil), which exert greater cardiac effects, particularly on the sinoatrial and atrioventricular nodes, leading to heart rate reduction (negative chronotropy).

**Figure 3:**
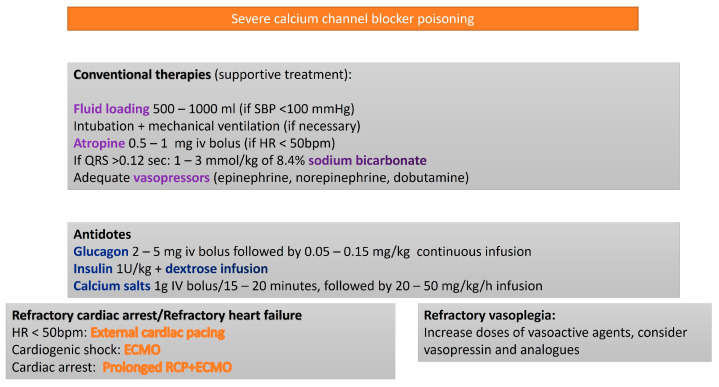
Algorithm for the treatment of severe calcium channel blocker poisoning.

While these pharmacodynamic differences are evident at therapeutic doses, they become less pronounced in overdose scenarios, potentially leading to severe shock characterized by bradycardia, vasodilation, or compromised myocardial contractility. Given the frequent use of sustained release formulations (e.g., diltiazem, verapamil, nifedipine) or the long half-life of certain agents (e.g., amlodipine), the toxic effects of CCBs may persist for prolonged periods, making them a major cause of poisoning-related mortality. Management typically involves atropine [19], calcium supplementation [20], vasopressors [1], high-dose insulin therapy [21], nitric oxide inhibitors (e.g., methylene blue) [22], and intravenous lipid emulsion (ILE) therapy [23] (Figure 3).

For intractable cases, V-A ECMO serves as a salvage therapy [24,25]. However, randomized control trials evaluating these treatments in cardiac arrest or refractory shock are lacking, and clinical recommendations are based on lower quality evidence from severely poisoned patients.

Charlotte E Goldfine et al. [26] conducted a comprehensive review of the clinical manifestations, diagnostic approach, and therapeutic strategies for CCB and BB toxicity. The authors emphasize the importance of early recognition and intervention, due to the potential for rapid hemodynamic deterioration. They discuss various pharmacologic treatments and explore the role of ECMO in refractory cases. ECMO has demonstrated efficacy in poisoned patients by providing hemodynamic support and gas exchange, allowing sufficient time for xenobiotic metabolism in cases of cardiogenic or mixed shock. A review of the outcomes in cardiovascular drug toxicity cases with early ECMO reported improved survival rates, supported by additional case reports [15,27,28,29,30,31], including in pediatric populations [32].

Daniel Finn et al. [8] conducted a literature review assessing the outcomes associated with ECMO support in CCB toxicity. Their findings suggest that ECMO can be a valuable intervention in refractory cases, providing hemodynamic stabilization and supporting recovery in patients unresponsive to conventional therapies. However, they highlight the need for further research to establish standardized protocols and define the patient population most likely to benefit from ECMO for CCB overdose.

Voiciu et al. [6] further advocate for ECLS in select cases of CCB intoxication. They emphasize that ECMO should not be withheld solely based on a prolonged cardiopulmonary resuscitation (CPR) duration, as survival has been documented even after 180 min of resuscitation.

The suggested criteria for ECMO initiation in CCB-related shock include persistent cardiac dysfunction despite catecholamine therapy (LVEF < 40–50%), high-dose vasopressor requirements (epinephrine + norepinephrine > 3 mcg/kg/min), and elevated blood lactate concentration (>8 mmol/L).

### 3.3. Local Anesthetics

Local anesthetics (LAs) exert their effect by reversibly inhibiting voltage-gated sodium channels, thereby disrupting nerve impulse transmission and abolishing pain perception. In cases of LA toxicity, patients may develop a syndrome known as local anesthetic systemic toxicity (LAST), characterized by a spectrum of neurological and cardiovascular manifestations. Central nervous system involvement, observed in the majority of LAST cases, frequently presents with seizures, but may also include agitation, syncope, dysarthria, perioral numbness, confusion, altered consciousness, and dizziness [8]. Although less common, cardiovascular toxicity poses a significant risk with documented occurrences of asystole and life-threatening arrhythmias, such as ventricular fibrillation or ventricular tachycardia [33].

**Figure 4:**
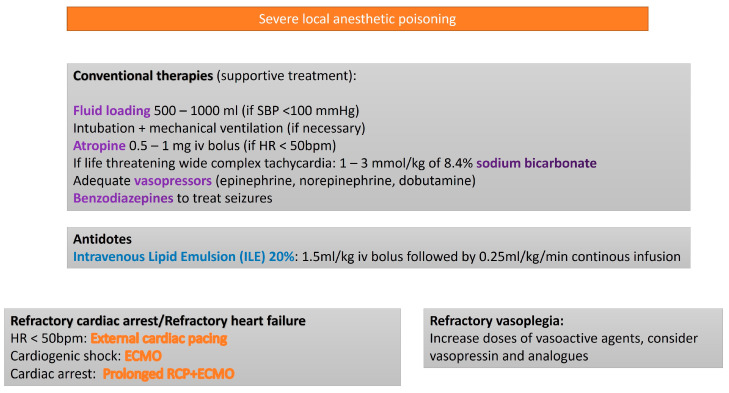
Algorithm for the treatment of local anesthetic poisoning.

The degree of toxicity among LAs varies, largely depending by the lipophilicity of their molecular structure. Bupivacaine, due to its high affinity and prolonged binding to cardiac sodium channels, demonstrates a markedly greater cardiotoxic potential than ropivacaine or lidocaine in preclinical models. Additionally, bupivacaine has been implicated in the induction of re-entry arrhythmias, conduction pathway suppression, and calcium channel blockade [34]. Experimental models have highlighted the exacerbating effects of hypoxia and acidemia on bupivacaine toxicity, underscoring the necessity of prompt ventilatory support and acid–base correction [35]. Many documented cases of LAST have occurred in perioperative settings, where early airway management and standard advanced life support (ALS) measures have facilitated the restoration of spontaneous circulation, often in the absence of intravenous lipid emulsion (ILE) therapy [36]. Nevertheless, the adjunctive administration of ILE has shown efficacy in both animal studies and clinical reports, with observational data supporting its role alongside ALS protocol [36,37]. Additional intervention, including hypertonic sodium bicarbonate solutions [38] and extracorporeal life support (such as V-A ECMO), have been utilized in severe cases, though their clinical benefit remains uncertain [39,40] (Figure 4).

In their Danish review, Johansson et al. [41] assert that, due to the short interval between exposure and symptom onset, severe circulatory impairment or cardiac arrest may be the first manifestation of toxicity [42]. Cardiopulmonary resuscitation should be initiated immediately and continued for an extended duration, potentially hours, due to the prolonged redistribution of the local anesthetic away from the affected organs and its concurrent metabolism and elimination. Sodium bicarbonate administration is recommended and, in cases of ongoing circulatory failure, the early consideration of extracorporeal circulatory support is crucial, as the condition is fundamentally reversible.

Attempts to enhance local anesthetic elimination through hemofiltration or dialysis have demonstrated minimal efficacy and should be avoided in the acute phase [41].

Bacon et al. [43] describe a case report of severe LAST following the administration of 36 mg/kg of topical lidocaine prior to transesophageal echocardiography, resulting in cardiac arrest that was resistant to lipid emulsion therapy. The patient was successfully resuscitated with VA-ECMO, highlighting the potential role of ECLS in managing severe LAST.

However, caution must be exercised regarding the simultaneous use of ILE and VA-ECMO. In a case series, Lee et al. [44] describe the potential effects of combining ILE and VA-ECMO concluding that the concurrent use can be associated with layering and agglutination in the tubing and circuits, cracking of stopcock valves, and increased frequency of blood clots within the circuits.

If combined, therapy with ILE and VA-ECMO is selected for the management of LAST, although the close monitoring of circuit parameters is recommended. This includes inspection for clot formation and occlusion, as well as monitoring machine indicators of potential malfunction, such as pressure drops, increased membrane resistance and pressures at the arterial and venous cannulae, and the oxygenator inlet [45].

### 3.4. Sodium Channel Blockers (SCBs)

Sodium channel blockers (SCBs), classified as Class I antiarrhythmic agents [2], can also be mimicked by other toxic substances when ingested in high doses. These substances, including antimalarials, opioids, venlafaxine, cocaine, carbamazepine, tricyclic and tetracyclic antidepressants, serotonin reuptake inhibitors, and certain antiepileptic drugs, induce SCB-like effects by inhibiting neuronal sodium channels [2,46]. The toxicity of these agents impedes myocardial cell depolarization, prolonging the QRS duration. This leads to a negative inotropic effect and increases the risk of fatal arrhythmias (e.g., ventricular tachycardia or fibrillation) [2]. Individuals with genetic predispositions, such as those with Brugada syndrome, are at elevated risk for arrhythmias during diagnostic challenges using intravenous Class I antiarrhythmic agents, sometimes requiring extracorporeal life support for electrical storms [47].

SCB toxicity manifests as QRS complex prolongation, hypotension, seizures, ventricular arrhythmias, and cardiovascular collapse. Several SCBs also influence other cardiac receptors and ion channels, complicating their management [2]. Although tricyclic antidepressant (TCAs) are the most well-characterized sodium channels blockers, numerous other substances cause similar life-threatening effects in overdose situations. Most treatment guidelines for such poisoning are derived from TCA-related studies. The recommended approach includes sodium bicarbonate, typically administered intravenously in hypertonic solutions (1000 mEq for adults, 500 mEq for children), to induce alkalemia, which has shown variable efficacy in case reports and animal models [48]. Other interventions, such as Class Ib antidysrhythmics (e.g., lidocaine [49] or phenytoin [50]) and intravenous lipid emulsion (ILE) [51,52], are suggested for managing the cardiotoxic effects of sodium channel blockers. However, the use of sodium bicarbonate, benzodiazepines for seizures, magnesium for wide complex tachycardia, and high-dose glucagon for hypotension remains insufficiently supported by clinical evidence to provide recommendations for their routine use.

**Figure 5:**
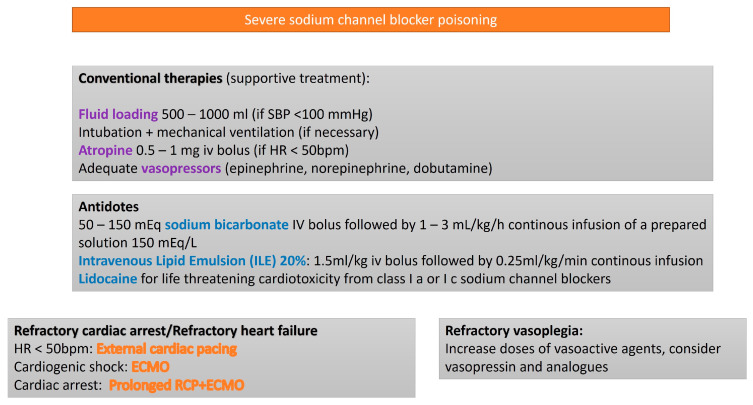
Algorithm for the treatment of sodium channel blocker poisoning.

ECMO has been successfully used to manage refractory cardiogenic shock caused by sodium channel blocker toxicity (Figure 5).

However, current evidence is limited to case reports and small case series [53,54], with no controlled observational studies or clinical trials available to confirm its efficacy. A recent case report [55] details the successful application of ECLS and hemoadsorption in managing severe venlafaxine intoxication necessitating cardiopulmonary resuscitation. The patient, after ingesting a substantial amount of venlafaxine, experienced profound cardiogenic shock and was unresponsive to conventional therapies. The implementation of VA-ECMO provided hemodynamic stabilization, while concurrent blood purification therapy facilitated the removal of the drug from circulation. This combined approach underscores the potential efficacy of integrating ECLS and hemoadsorption in the case of life-threatening intoxications with agents like venlafaxine.

In their study, Voiciu et al. [6] also discuss the indications for ECLS in SCB-related cardiogenic shock, noting that the optimal cutoff values for all parameters remain undetermined. The proposed criteria include cardiac dysfunction despite catecholamine therapy, with LVEF < 35 %, combined epinephrine and norepinephrine dose > 1 mcg/kg/min, lactate concentration > 5 mmol/L, and QRS duration > 150 ms.

### 3.5. Sympathomimetics

Sympathomimetic toxicity results from excessive adrenergic activation, typically induced by substances such as amphetamines, cathinones, and certain synthetic cannabinoid receptor agonists. In clinical practice, the specific agent involved is often unidentified, necessitating a symptom-driven management approach. The pathophysiology of sympathomimetic poisoning is primarily attributed to excessive catecholamine release, leading to heightened metabolic and psychomotor activity. Clinical manifestations vary in severity and may include tachycardia, hypertension, agitation, seizures, hyperthermia, rhabdomyolysis, and metabolic acidosis [56,57].

Acute cardiovascular events, including sudden cardiac arrest, may present as ventricular fibrillation, ventricular tachycardia, or pulseless electrical activity [58,59]. Coronary vasospasm can precipitate myocardial infarction even in individuals with angiographically normal coronary arteries. Additionally, stress-induced cardiomyopathy (Takotsubo) has been reported, which, despite its potential severity may resolve spontaneously in survivors [60]. Hyperthermia is a critical and rapidly progressive complication [58]. While physical restraints may be temporarily necessary, prolonged use can exacerbate agitation and hyperthermia. Despite multiple studies comparing sedative agents for severe psychomotor agitation, no clinical trials have specifically addressed the strategies for preventing or managing cardiac arrest in this context. Consequently, therapeutic recommendations are largely derived from case reports, expert consensus, and experimental models. Although no specific antidote exists for sympathomimetic toxicity, adequate sedation is essential to mitigate agitation and prevent secondary complications such as delirium, rhabdomyolysis, and hyperthermia [61]. In certain cases, high-dose sedation is required [62]. External cooling plays a pivotal role in controlling hyperthermia and potentially mitigating neurologic and systemic organ injury [63]. Effective sedation often reduces the need for antihypertensive therapy; however, evidence guiding the management of severe cardiovascular toxicity refractory to sedation remains limited. Agents such as alpha-1 receptor antagonists, alpha-2 receptor agonists, calcium channel blockers, nitrates, and mixed alpha–beta blockers have been employed for tachycardia and hypertension, but no standardized protocol has been established [64].

Mechanical circulatory support, including V-A ECMO [60,65] and intraortic balloon pump (IABP) [66], has been successfully used in cases of cardiogenic shock secondary to stress-induced cardiomyopathy. Although Takotsubo cardiomyopathy can be life threatening, it frequently resolves spontaneously within days to weeks with appropriate hemodynamic support.

Notably, several case reports highlight the utility of ECMO in managing of cardiogenic shock induced by sympathomimetic toxicity.

Grimm et al. [67] describe a case of cocaine-induced myocardial infarction due to coronary vasospasm. While vasodilators are commonly used in treatment, they may exacerbate the loss of cardiovascular homeostasis in critically ill patients. In this case, extracorporeal membrane oxygenation provided vital circulatory support, facilitating myocardial recovery after recurrent ischemic injury and arrhythmias.

Similarly, De Vroey et al. [68] report a case of a young polydrug user who developed acute heart failure following initial cocaine use. Despite intensive medical treatment, he remained hemodynamically unstable, necessitating ECMO, which ultimately led to complete left ventricular recovery.

Oredegebe et al. [69] describe a 26-year-old male with Graves’ disease and cocaine abuse who developed Takotsubo cardiomyopathy and cardiogenic shock due to a thyroid storm. Advanced circulatory support, including ECMO, alongside antithyroid therapy and corticosteroids, facilitated full myocardial recovery, allowing for successful weaning from vasopressors and mechanical support.

### 3.6. Intoxication from Other Cardiotoxic Drugs

Virtually any drug or toxin capable of inducing cardiogenic shock, malignant arrhythmias, or cardiac arrest may warrant V-A ECMO support. Numerous case reports document successful V-A ECMO utilization in drug-induced cardiogenic shock.

Schreiber et al. [70] describe a case of severe cardiogenic shock following yew (Taxus baccata) poisoning, successfully managed with V-A ECMO. The ingestion of yew plant material led to profound circulatory instability that was unresponsive to conventional therapies. V-A ECMO provided critical circulatory support, enabling metabolic stabilization and cardiac recovery. This case underscores the high lethality of yew poisoning due to its potent cardiotoxic effects and highlights ECMO as a life-saving intervention in toxin-induced cardiogenic shock. Similarly, Daniels Z. et al. [71] report a pediatric case of severe cardiotoxicity following yew ingestion, successfully treated with V-A ECMO.

Vijayakumar et al. [72] describe an adult male who intentionally ingested aluminum phosphide (ALP) in a suicide attempt. ALP poisoning is associated with high mortality due to the release of phosphine gas, which induces severe mitochondrial dysfunction, leading to persistent myocardial depression, hypotension, metabolic acidosis, and acute respiratory distress syndrome (ARDS). In this case, the patient presented with cardiovascular collapse and was promptly initiated on ECMO in the emergency department. This intervention provided critical hemodynamic support, facilitating metabolic stabilization and cardiac recovery.

Chen et al. [73] report a case of severe cardiogenic shock following the ingestion of Macleaya cordata, a plant containing toxic alkaloids. The patient developed profound circulatory instability that was unresponsive to conventional therapy. V-A ECMO was successfully employed to maintain circulation, allowing for metabolic stabilization and myocardial recovery.

Okuda et al. [74] describe a case of life-threatening intoxication following the ingestion of an oleander leaf decoction. Oleander contains potent cardiac glycosides, such as oleandrin, which can induce severe arrhythmias and cardiac arrest. ECMO was successfully implemented, providing essential circulatory support and enabling cardiac recovery.

Certain chemotherapeutic agents and monoclonal antibodies can also provoke fatal arrhythmias and cardiogenic shock.

Rundhawa et al. [75] present a case of severe myocarditis following entrectinib administration, a tyrosine kinase inhibitor used in oncology. The patient experienced acute cardiac dysfunction with significant hemodynamic compromise, necessitating V-A ECMO for circulatory support, ultimately leading to myocardial recovery.

Similarly, Torre et al. [45] report a case of life-threatening ventricular tachyarrhythmias induced by ibrutinib, a tyrosine kinase inhibitor used in hematologic malignancies. The arrhythmic storm was refractory to conventional antiarrhythmic therapy, prompting V-A ECMO initiation. Additionally, ILE therapy, which acts as a lipid sink for lipophilic toxins, was administered, leading to successful patient stabilization.

Rateesh et al. [76] describe a case of acute heart failure following the administration of 5-fluorouracil (5-FU), a chemotherapeutic agent with known cardiotoxic potential. Despite intensive medical management, the patient developed severe hemodynamic decompensation requiring V-A ECMO, which ultimately facilitated myocardial recovery.

Shah et al. [77] report a case of severe acute heart failure following high-dose methotrexate therapy. The patient experienced profound circulatory collapse, which was resistant to conventional treatments, and was successfully stabilized with V-A ECMO.

In addition, acute organophosphate pesticide poisoning (AOPP) can induce severe cardiac toxicity. Organophosphates and carbamates, commonly found in pesticides, exert their toxic effect by irreversibly inhibiting acetylcholinesterase, leading to excessive cholinergic stimulation. Clinical manifestation includes muscarinic symptoms (bradycardia, bronchospasm, bronchorrhea, miosis, hypersalivation, lacrimation, gastrointestinal hypermotility, and diaphoresis), nicotinic effects (tachycardia, mydriasis, and muscle fasciculations progressing to neuromuscular blockade and paralysis), and central nervous system involvement (altered mental status, apnea, and seizures). Organophosphates irreversibly inhibit acetylcholinesterase via covalent binding, leading to enzyme aging and permanent inactivation. In contrast, carbamates inhibit the enzyme transiently, with spontaneous dissociation enabling reactivation. Prompt intervention is critical to prevent progression to respiratory failure and cardiac arrest. Management includes decontamination (with copious water and soap), pharmacological reversal, and supportive care. Atropine effectively counteracts muscarinic overstimulation but does not reverse the nicotinic effect, necessitating adjunctive benzodiazepines for seizures prophylaxis and management. Oximes, if administered before enzymatic aging occurs, can restore acetylcholinesterase activity and improve neuromuscular function, though their efficacy may be organophosphate-specific.

Yang li et al. [78] describe a severe organophosphate poisoning case resulting in myocardial injury and advanced cardiogenic shock. Despite maximal medical therapy, the patient showed ongoing hemodynamic deterioration necessitating V-A ECMO for circulatory support, which ultimately facilitated metabolic stabilization and cardiac recovery.

## 4. Discussion

Extracorporeal membrane oxygenation (ECMO), an advanced extracorporeal life support (ECLS) modality, provides temporary circulatory and/or respiratory assistance in cases of severe cardiac and/or pulmonary failure. By partially or completely replacing native circulation and gas exchange, ECMO serves as a bridge to recovery, transplantation, or short-term mechanical support in critically ill patients. An ECMO circuit consists of a closed loop system incorporating a venous drainage cannula, centrifugal pump, oxygenator, gas blender, heat exchanger, and return cannula (venous return cannula in veno-venous ECMO, arterial cannula in veno-arterial ECMO). The choice of cannulation strategy is dictated by the underlying pathology.

Veno-arterial ECMO is an advanced form of mechanical circulatory support that operates in parallel with the native cardiopulmonary system. In this configuration, oxygenated blood is reinfused directly into the arterial circulation, effectively bypassing the failing heart.

Although V-A ECMO is a temporary supportive intervention, optimal outcomes depend on careful patient selection, tailored cannulation strategies, and vigilant management to mitigate complications. As the utilization of this advanced circulatory support modality continues to expand, a thorough understanding of its indications is essential to optimize clinical efficacy, refine prognostic expectations, and minimize associated risks. The ELSO guidelines, authored by Lorusso et al. [79], outline the indications for V-A ECMO implementation. Short-term mechanical circulatory support should be considered in patients with severe myocardial failure with circulatory collapse due to a potentially reversible or surgically correctable etiology. Clinical scenarios warranting V-A ECMO include both the medical and post-surgical causes of acute cardiogenic shock (acute coronary syndrome [80], fulminant myocarditis, cardiotoxic drug poisoning, acute anaphylaxis, sepsis-induced cardiomyopathy, refractory arrhythmias, end-stage dilated or ischemic cardiomyopathy, refractory hypothermia with cardiovascular instability, and massive pulmonary embolism) [81]. Additionally, V-A ECMO is utilized in post-cardiac surgery settings, including post transplantation cases where conventional therapies fail to restore hemodynamic stability [82].

Furthermore, V-A ECMO can be deployed in cardiopulmonary resuscitation (E-CPR) for refractory cardiac arrest [83] and as a prophylactic strategy for high-risk percutaneous cardiac interventions.

The optimal scenario for V-A ECMO initiation arises when conventional medical therapy, including fluid resuscitation, inotropic support and, if applicable, intra-aortic balloon pump (IABP), fails to restore hemodynamic stability. Ideally, ECMO deployment should occur before the onset of multiorgan dysfunction and should be guided by a comprehensive echocardiographic assessment. Patient selection should incorporate factors such as age, comorbid conditions, and the prognosis of the underlying pathology. However, advanced age alone should not serve as an absolute contraindication, particularly in cases where meaningful cardiac recovery is anticipated [25,84].

This review explores the role of V-A ECMO and its applicability in cases of poisoning due to cardiotoxic drugs.

Drug-induced cardiovascular failure represents the primary cause of mortality in severe acute intoxications. In cases of refractory shock or cardiac arrest that is unresponsive to optimal conventional therapies, advanced interventions such as ECLS may be warranted. V-A ECMO serves as an advanced resuscitative strategy offering both cardiac and pulmonary support. In cases of poisoning, V-A ECMO mitigates persistent cardiogenic shock by providing mechanical circulatory assistance while facilitating toxin clearance. The use of V-A ECMO in toxicological emergencies has been steadily increasing [13,85]; however randomized controlled trials (RCTs) comparing its efficacy to standard supportive care in poisoned patients are still lacking. Observational studies suggest that poisoned patients requiring V-A ECMO for cardiac arrest or refractory shock exhibit lower mortality compared with both non-toxicological ECMO patients and those receiving conventional intensive care and antidotal therapy alone [15,85]. This favorable prognosis likely stems from the reversible nature of most drug intoxications, provided irreversible end organ damage has not occurred, allowing spontaneous recovery via renal, hepatic, or extracorporeal toxin elimination.

The application of V-A ECMO in toxicological emergencies is constrained by factors such as resource availability, transport logistics, patient comorbidities, and procedural risks. A nuanced assessment of both the toxicokinetics of the ingested substance and the patient’s clinical status is essential when considering V-A ECMO initiation. Notably, V-A ECMO does not address distributive shock or mitigate direct cellular toxicity. A multidisciplinary approach, incorporating expertise from medical toxicologists or poison control centers, is recommended to optimize patient selection [86]. When employed during cardiac arrest, V-A ECMO contributes to extracorporeal cardiopulmonary resuscitation (E-CPR). Retrospective analyses have identified cardiotoxic poisoning as an independent predictor of survival among V-A ECMO patients with cardiac arrest or refractory shock [87]. Observational studies further suggest that V-A ECMO reduces mortality in poisoning-related cardiovascular collapse, with reported survival rates varying based on the etiology of intoxication [15,24,85,88]. However, complications such as limb ischemia, hemorrhage, stroke, and infection remain significant concerns [89]. For patients with persistent non-perfusing dysrhythmias, V-A ECMO facilitates forward circulatory flow, enabling toxin clearance. Case reports document successful V-A ECMO utilization in poisoned individuals with sustained arrhythmias [90,91]. Nonetheless, in cases involving severe metabolic or hematological toxicities, V-A ECMO has been associated with higher mortality rates [84]. Moreover, its efficacy remains uncertain in poisonings characterized by profound vasodilatory shock with preserved cardiac function, direct cellular toxicity, impairment of oxidative metabolism, or universally fatal toxic syndrome that is unresponsive to temporary circulatory support. Given these limitations, it is essential to consider alterative mechanical circulatory support (MCS) strategies tailored to the specific pathophysiology of drug-induced cardiotoxicity. While V-A ECMO remains the most comprehensive modality for circulatory support in cases of severe myocardial depression and biventricular failure, devices such as the intra-aortic balloon pump (IABP) and Impella may offer selective advantages specific clinical scenarios [92,93,94,95,96,97,98]. IABP can be employed in less severe cases or as an initial bridging strategy due to its ease of use and availability; however, its hemodynamic support is often insufficient in profound shock, necessitating escalation to V-A ECMO. Impella, although not widely available during the earlier years of the reviewed timeframe, now represents a valuable option in selected cases of isolated left ventricular failure with preserved right ventricular function. Furthermore, it may serve as a step down support strategy following initial stabilization with V-A ECMO, provided that right ventricular recovery is complete and pulmonary circulation remains adequate. Nevertheless, in cases of drug-induced biventricular failure or refractory cardiac arrest, V-A ECMO continues to be the only viable life-saving intervention, capable of delivering full cardiopulmonary support until myocardial recovery and clearance of the toxic insult are achieved.

## 5. Conclusions

The application of veno-arterial ECMO in the management of cardiotoxic drug intoxications represents a critical intervention in life-threatening scenarios where conventional therapeutic measures fail to provide adequate hemodynamic support. Despite substantial advancements in ECMO technologies and techniques, its role remains highly specialized and context-dependent. In cases of acute cardiovascular collapse due to cardiotoxic substances, V-A ECMO can restore systemic circulation, optimize end-organ perfusion, and facilitate drug clearance, thus enabling the reversal of the toxic effects when combined with appropriate pharmacological and supportive treatments. However, the decision to initiate V-A ECMO should be guided by careful patient selection, considering factors such as the severity of intoxication, the potential for drug metabolism, and the prognosis of the underlying toxicological insult. The early recognition of the need for extracorporeal support, along with expertise in ECMO circuit management, is paramount for achieving favorable outcomes. While promising, the clinical evidence surrounding V-A ECMO in cardiotoxic drug intoxication remains limited and warrants further investigation. Prospective multicenter studies and well-designed clinical trials are essential to refine patient selection criteria, optimize management protocols, and better understand the long-term outcomes of ECMO-assisted recovery in this specialized area of toxicology. Additionally, future advancements in ECMO technology and adjunctive therapies could further enhance the efficacy of this life-saving modality in the context of drug-induced cardiovascular toxicity.

## Figures and Tables

**Table 1:** Overview of cardiotoxic agents treated with ECLS: indications and reported survival outcomes from the literature.

Toxic Agent	Indication Criteria for ECMO	Reported Survival Outcome
***Beta blockers***	Persistent cardiogenic shock despite maximal inotropic and vasopressor support or evolution to cardiac arrest	63.6% [7]
***Calcium channel blockers***	Persistent cardiogenic shock despite maximal inotropic and vasopressor support or evolution to cardiac arrest	84.6% [8]
***Local anesthetics***	Persistent cardiogenic shock despite maximal inotropic and vasopressor support or evolution to cardiac arrest due to local anesthetic systemic toxicity (LAST) unresponsive to conventional resuscitation and lipid emulsion therapy	Survival reported in case series and isolated reports; exact rate not quantifiable due to rarity of cases
***Sodium channel blockers***	Persistent cardiogenic shock despite maximal inotropic and vasopressor support or evolution to cardiac arrest	Survival reported in case series and isolated reports; exact rate not quantifiable due to rarity of cases
***Sympathomimetics***	Cardiac arrest unresponsive to advanced resuscitation	Favorable outcomes in isolated cases; no consistent survival rate reported.
***Other cardiotoxic drugs***	Persistent cardiogenic shock despite maximal inotropic and vasopressor support or evolution to cardiac arrest	Favorable outcomes in isolated cases; no consistent survival rate reported.

## Abbreviations

The following abbreviations are used in this manuscript:

ECLS	Extracorporeal life support
V-A ECMO	Veno-arterial Extracorporeal membrane oxygenation
BBs	Beta blockers
CCBs	Calcium Channel blockers
SCBs	Sodium Channel blockers
LAs	Local Anesthetics
ILE	Intravenous Lipid Emulsion
RCP	Cardiopulmonary Resuscitation
LVEF	Left ventricular ejection fraction
